# Controlled Localized Metal–Organic Framework Synthesis on Anion Exchange Membranes

**DOI:** 10.1021/acsami.4c02882

**Published:** 2024-06-10

**Authors:** Harm T. M. Wiegerinck, Özlem H. Demirel, Harmen J. Zwijnenberg, Tom van der Meer, Timon Rijnaarts, Jeffery A. Wood, Nieck E. Benes

**Affiliations:** Membrane Science and Technology Cluster, MESA+ Institute for Nanotechnology, University of Twente, 7500AE Enschede, The Netherlands

**Keywords:** MOF coating, Donnan exclusion, counter-diffusion synthesis, anion exchange membrane, Cu-BTC, ZIF-8

## Abstract

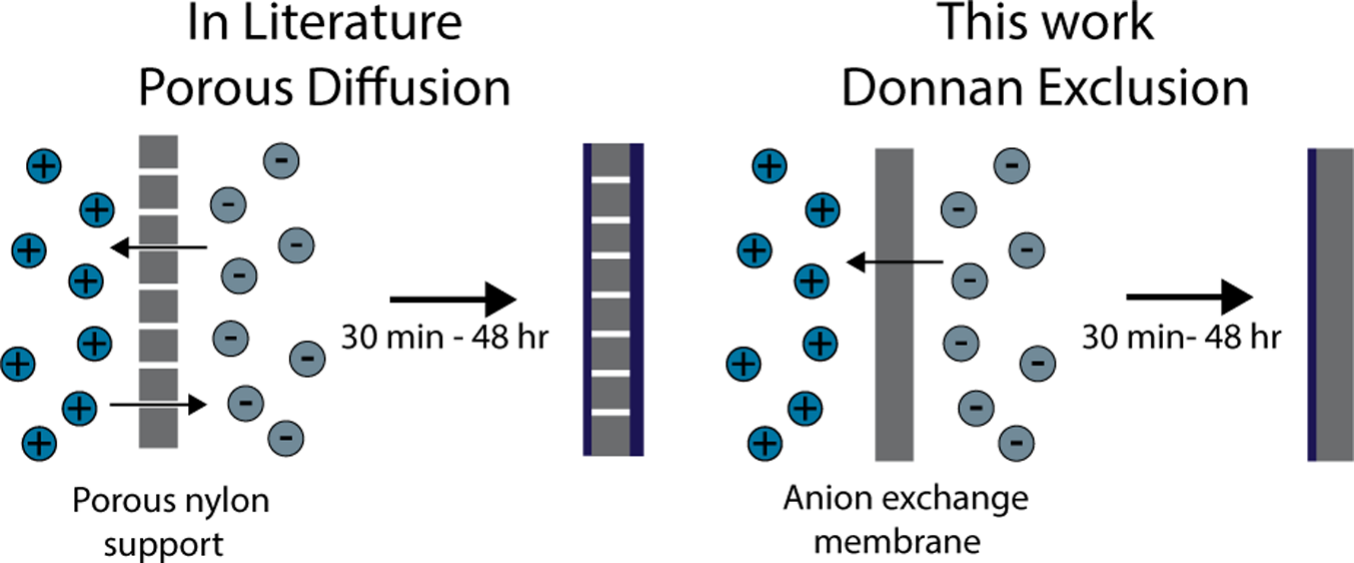

Metal–organic framework (MOF) films can be used in various applications. In this work, we propose a method that can be used to synthesize MOF films localized on a single side of an anion exchange membrane, preventing the transport of the metal precursor via Donnan exclusion. This is advantageous compared to the related contra-diffusion method that results in the growth of a MOF film on both sides of the support, differing in quality on both sides. Our proposed method has the advantage that the synthesis conditions can potentially be tuned to create the optimal conditions for crystal growth on a single side. The localized growth of the MOF is governed by Donnan exclusion of the anion exchange membrane, preventing metal ions from passing to the other compartment, and this leads to a local control of the precursor stoichiometry. In this work, we show that our method can localize the growth of both Cu-BTC and ZIF-8 in water and in methanol, respectively, highlighting that this method can used for preparing a variety of MOF films with varying characteristics using soluble precursors at room temperature.

## 1 Introduction

Metal–organic frameworks (MOFs) are a class of nanoporous materials consisting of metal centers connected with organic ligands.^1^ Their chemical and thermal stability, high surface area, and porosity have attracted interest in many different application fields ranging from electronic devices and catalysts to batteries and membranes.^2−4^ Early studies have mostly focused on obtaining MOF powders.^5−8^ However, with the application spectrum moving more toward film-based sensors and separation membranes, more focus is put on the preparation of MOF films.^9^ Different techniques have been utilized to form thin MOF films on various substrates such as in situ synthesis,^10−12^ secondary growth,^13−15^ microwave irradiation,^16,17^ layer-by-layer coating,^18,19^ dip coating,^20,21^ electrochemical synthesis,^22^ counter-diffusion,^23−25^ and interfacial synthesis.^26−29^

A relatively simple MOF film formation method was proposed by Yao et al.^23^ They used the principle of counter-diffusion applied to ZIF-8 synthesis on a flexible porous polymer (nylon) support.^23^ In this method, the two precursors dissolved in methanol were separated by the support material for the MOF film. Due to the concentration gradients, both precursors diffuse through the support to the other side. This is referred to as counter-diffusion. The precursors react, resulting in the formation of ZIF-8 films on both sides of the support. The nucleation and growth of ZIF-8 and MOFs, in general, depend on the local molar ratio of precursors.^30,31^ Therefore, different ZIF-8 morphologies were obtained on both sides. On the metal solution side, the ratio of organic ligand to metal ion approached zero, resulting in the formation of a thicker layer of larger crystalline domains. On the organic ligand solution side, the local molar ratio was larger than the molar ratio of the precursor solutions, where the MOF crystallized into a thinner layer of smaller crystal domains.^23^

**Figure 1:**
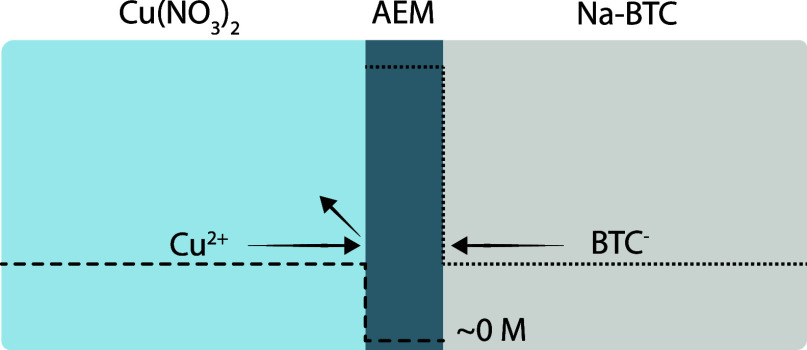
Concentration profiles of copper cations and trimesic acid anions in contact with an AEM.

While this method is successfully applied for coating different supports and can be used for several types of MOFs,^23,32−34^ this film synthesis method could possibly be improved significantly by localizing the MOF formation solely to one side of the support with a high ligand-to-metal ratio to ensure that the crystal growth conditions are more favorable for forming high-quality MOF films, while the amount of precursor that is consumed during the synthesis is reduced. In theory, this can be accomplished by blocking the transport of the metal precursor through the support and enriching the organic ligand in the support. In most MOF syntheses, the metal precursor is ionic and positively charged, while the organic ligand is either negatively charged or neutral. This opens up an opportunity to block the transport of the metal precursor based on its charge. Anion exchange membranes (AEMs) are a potential support that could block the metal precursor. Ion exchange membranes are a class of membranes that have a large number of strongly dissociating ionic groups in their structure, for example, positively charged quaternary ammonium groups in case of AEMs that dissociate strongly in water. Due to the presence of a large amount of fixed charges inside the membrane, negative ions can pass through this membrane, while positive ions are strongly repelled and therefore excluded. This phenomenon is referred to as Donnan exclusion,^35^ illustrated schematically for Cu-BTC formation in Figure 1. Furthermore, Donnan exclusion results in an enrichment of organic counterions in the AEM equal to the number of ionic groups. In water, the fixed charge density and therefore the amount of charged counterions are typically on the order of 1 M. This enrichment leads to a larger organic-to-metal precursor ratio as compared to a neutral support counter-diffusion method close to the membrane surface on the metal ion solution side.

In this work, we first demonstrate the localized MOF synthesis method with an AEM support and the MOF precursors dissolved in water, meaning that the membrane is highly selective and the charged precursors can fully dissociate. The model MOF that we selected for this study is Cu-BTC. However, not all MOFs are compatible with water. To assess the robustness of our proposed method, the solvent was changed. As an alternative, we selected methanol since, while it has a lower dielectric constant, it is still relatively high compared to that of other organic solvents. This will potentially result in a lower membrane selectivity. The localized MOF synthesis in methanol is also explored by synthesizing ZIF-8 in this medium.

## 2 Experimental Methods

### 2.1 Chemicals

Zinc nitrate hexahydrate (99%; Sigma-Aldrich), copper nitrate trihydrate (99–104%; Sigma-Aldrich), 2-methylimidazole (HMIM) (99%; Sigma-Aldrich), trimesic acid (BTC) (98% Sigma-Aldrich), sodium hydroxide (NaOH) (99%; Sigma-Aldrich), sodium chloride (NaCl) (Sanal P; AkzoNobel), and methanol (>99%; Boomlab) were used without any further purification for preparing the MOFs and for various other experimental aspects.

### 2.2 MOF Precursor Preparation and Membrane Pretreatment

Cu-BTC is synthesized with trimesic acid (benzene 1,2,3-tricarboxylic acid) as the organic ligand and a copper salt such as copper nitrate. However, trimesic acid is poorly soluble in water. Therefore, trimesic acid was first converted to its sodium salt (Na-BTC) by following the method of Nowacka et al.;^36^ a stoichiometric amount of sodium hydroxide pellets was added to a BTC-water slurry while stirring, resulting in a clear Na-BTC solution of 0.1 M at a pH of around 10. We also performed a synthesis of Cu-BTC in methanol. For this synthesis, trimesic acid was used in its acid form because it is soluble in methanol. For every Cu-BTC synthesis, copper nitrate trihydrate was dissolved in water or methanol at a concentration of 0.1 M.

ZIF-8 was synthesized with HMIM (2-methylimidazole) and a zinc-containing salt such as zinc nitrate. In this case, both precursors fully dissolved in methanol without pretreatment. Zinc nitrate hexahydrate and HMIM were dissolved separately in methanol while stirring to obtain solutions of 0.05 M zinc nitrate and 0.4 M HMIM. Commercial AEMs (Fujifilm Type 1; Fujifilm Europe B.V.) were cut into 4 cm circles and were soaked for at least 24 h in Milli-Q water or methanol, matching the solvent used for the precursor solutions for both Cu-BTC and ZIF-8.

### 2.3 MOF Synthesis

In a diffusion cell setup (see Supporting Information Figure S10), the pretreated commercial Fujifilm AEM is put between the two glass reservoirs, which are then connected via tightening bolts. Next, the two reservoirs are filled with either the metal precursor or the organic precursor solution, and the reservoirs are closed to avoid evaporation. The setup is left for at least 30 min up to 48 h to explore the growth rate of the MOF films in more detail. Next, the reservoirs are emptied, and the membrane is removed by opening the setup. Finally, the coated membranes are immersed in the solvent used during the synthesis for several minutes to remove any residual precursor present on the surface of the membrane. Finally, the membrane is stored in a fresh amount of solvent used during the synthesis until SEM analysis.

### 2.4 SEM Analysis

To prepare the MOF-coated membranes for SEM analysis, coated membrane samples were cut out of the middle of the MOF-coated membrane, placed on a sample holder, and dried in a vacuum oven to remove the used solvent. Next, the sample was sputtered with a platinum/palladium coating of 5 nm. SEM (JSM-6010LA, JEOL) images of the surface were all made with the secondary electron intensity detector at a voltage of 5 kV and at various magnifications. EDS analyses were performed with a voltage of 15 kV, and the settings were adjusted for every EDS analysis to ensure that the electron count rate was above 2000. To estimate the thickness of Cu-BTC, some broken-off sections were analyzed by SEM. For the thickness estimate of the ZIF-8 coating, a cross-section of the membrane with the coating was made and analyzed with a field emission scanning electron microscope (JSM-7610F, JEOL), using the same procedure described above.

## 3 Results

**Figure 2:**
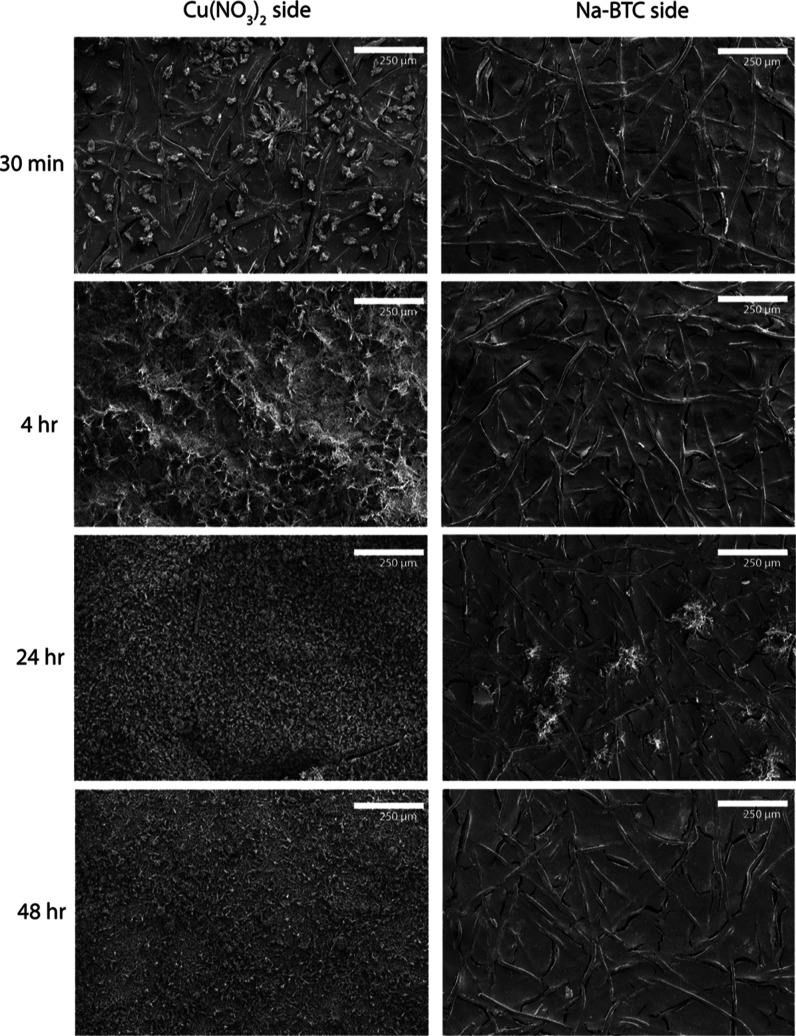
SEM images of the morphology of the surface of AEMs on the copper nitrate side and the Na-BTC side, for different synthesis times, magnified 100 times. The white scale bars indicate a distance of 250 μm.

In Figure 2, both sides of the membrane surface after Cu-BTC syntheses of different durations are shown. Here, it can be seen that on the CuNO_3_ side, after 30 min, small individual crystals appeared. After 4 h, a flaky crystal structure can be observed, and the underlying features of the membrane fibers are completely covered by the Cu-BTC. After 24 and 48 h, the surface seems less chaotic and more uniform compared to that of the 4 h sample. On the Na-BTC side, no consistent growth of crystals can be seen even after 48 h. The membrane also appeared to be cracked on the Na-BTC side. This is caused by vacuum drying and is only apparent on the Na-BTC side due to the empty surface on this side. These results show that the AEM effectively prevents the copper cations from transporting through the membrane by Donnan exclusion. However, because of the presence of hydroxide ions as both the native counterion in the membrane as well as in the synthesized Na-BTC solution of pH 10, the crystals that form on the membrane surface could well be copper hydroxide instead of the expected Cu-BTC. However, from the more detailed analysis presented in Supporting Information Section S1, it was concluded that primarily Cu-BTC has formed. Nevertheless, the presence of hydroxide ions can definitely affect the synthesis and the observed MOF morphology.

From these results, it can be concluded that AEM can be used to localize the MOF synthesis to a single side of the membrane. The stability as well as the solubility in water of typical precursors is limited; in addition, some MOFs are unstable in water.^37^ Furthermore, as discussed in Section S1 of the Supporting Information, the pH could have a pronounced effect on the morphology of the MOF. Therefore, an organic solvent should be used to expand the range of applicability to a wider range of MOFs.

A common solvent to use for MOF syntheses is methanol. In the past, it was shown by Shou and Tanioaka that although the charge density of ion exchange membranes is reduced about 7 to 11 times, the fixed charge concentration can still be on the order of 50 mM.^38^ Next to the charge in the membrane, also the dissociation of the ions is affected by the solvent to some extent. For instance, it can be derived from the equilibrium constants of Al-Baldawi et al.^39^ that Zn(NO_3_)_2_ dissociates primarily into ZnNO_3_^+^, while less than 10% remains uncharged at the concentrations used in this work (see Supporting Information Section S2). To show the potential of the localized MOF growth in methanol, ZIF-8 is synthesized. For ZIF-8 syntheses performed in water, a large amount of the zinc ions react with hydroxide ions and form considerable amounts of zinc hydroxide instead of ZIF-8, as shown previously in the literature^40^ as well as in our previous experiments (See Supporting Information Section S3). Therefore, methanol is more suitable for ZIF-8 synthesis compared to water.

**Figure 3:**
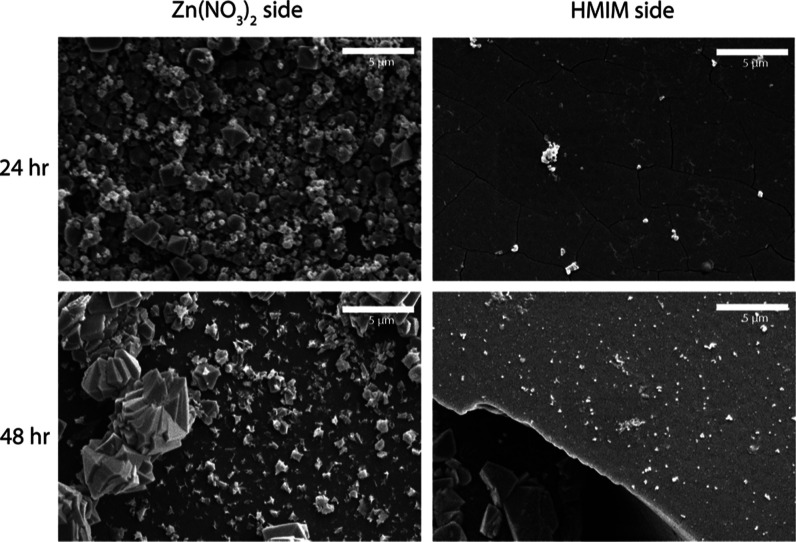
SEM images of the morphology of the surface of AEMs on the zinc nitrate side and the HMIM side, for different synthesis times, magnified 5000 times. The white scale bars indicate a distance of 5 μm.

**Figure 4:**
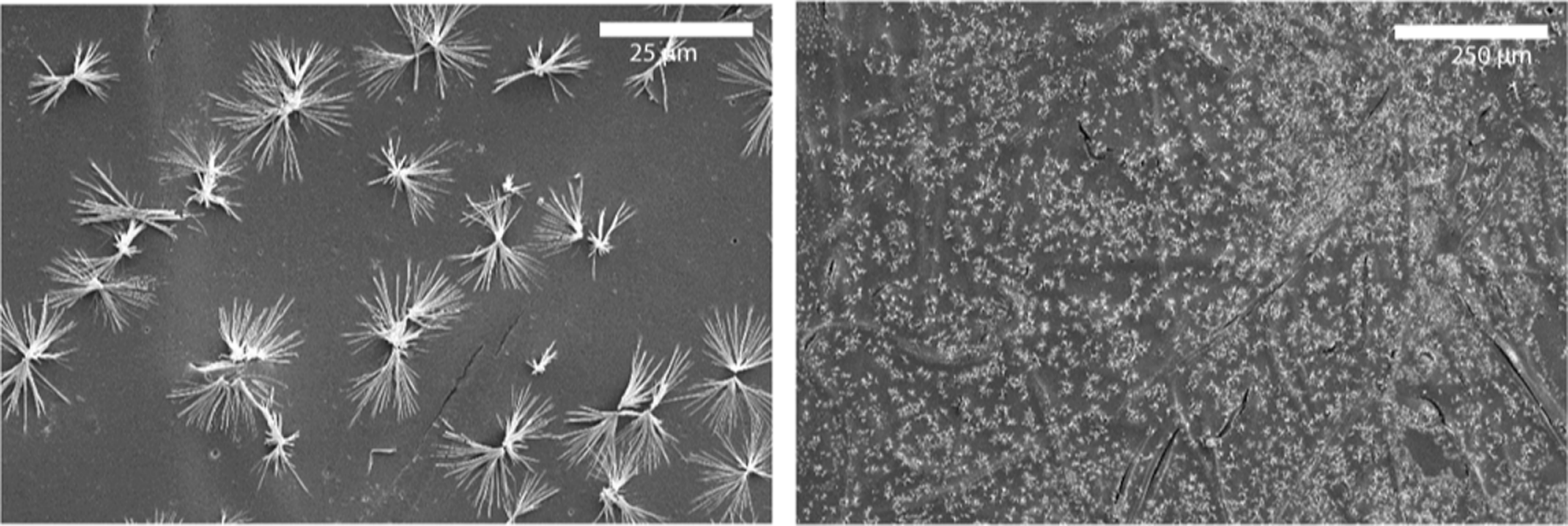
SEM image of the morphology of the surface of AEMs on the copper nitrate side for Cu-BTC prepared in methanol for a synthesis time of 4 h at a magnification of 1000 and 100 times, on the left and right, respectively. The white scale bars indicate a distance of 25 μm on the left and 250 μm on the right.

In Figure 3, the synthesis results of ZIF-8 in methanol can be observed. In this figure, it can be seen that after 24 h, the surface on the zinc nitrate side is covered in square ZIF-8 crystals with a wide size distribution, whereas after 48 h, there are smaller and larger crystals present, and the surface coverage is seemingly lower compared to the result after 24 h. On the HMIM side, there are small individual crystals present on the surface after 24 h and slightly more after 48 h, but relative to the zinc nitrate side, the amount on the HMIM side is almost negligible. It should also be noted that these images were made at a higher magnification compared to the Cu-BTC synthesis in water since the ZIF-8 layers were thinner compared to the Cu-BTC layers grown in water, as shown previously. This is also apparent from Figure S7 in the Supporting Information, where the contours of the reinforcing fibers can still be seen despite the ZIF-8 coating. From these results, it can be inferred that in methanol, the zinc cations are still prevented from passing the membrane by Donnan exclusion to a large extent. However, clearly, some transport of cations through the membrane has occurred, either due to the lower charge density of the AEM, reducing the permselectivity of the AEM, or due to the presence of neutral undissociated Zn(NO_3_)_2_ that can diffuse through the AEM. The seemingly lower surface coverage after 48 h can be attributed to the drying stresses of the sample preparation. When the crystals grow somewhat larger, the surface-to-volume ratio decreases, making them more prone to break off. Based on the results shown in Figures 2 and 3, the kinetics of crystallization appear faster in water compared to that in methanol as the ZIF-8 layer had to be observed under higher magnification and does not completely cover the fibrous features of the AEM (see also Section S4 of the Supporting Information). In both these cases, it is possible to form a MOF film on top of the AEMs after 24 h. The thickness was roughly estimated by observing film cross sections under SEM; this showed that the ZIF-8 film was approximately 0.5 μm, while the Cu-BTC film was 20 μm after 24 h. There could be various reasons for the difference in thickness. However, it is out of the scope of this work to find the main cause for the difference in crystallization kinetics. Therefore, we describe a few potential reasons. First, the charged nature of HMIM is unclear in methanol, and in case it is neutral, the concentration would not be enriched in the membrane as would be the case for BTC anions in water. Consequently, the crystallization proceeds slower. Second, the growing MOF entities could have a higher solubility in methanol, resulting in lower supersaturation and consequently slower growth of the crystals. Finally, the reaction between the precursors could be more hindered in methanol due to the less dissociated precursors. To further study the effect of the solvent on the MOF crystallization, Cu-BTC was also synthesized in methanol for 4 h (see Figure 4). In this figure, several small individual crystals can be seen to have formed on top of the membrane. This result is in strong contrast to the results obtained for Cu-BTC in water, where a Cu-BTC coating covered the membrane completely after 4 h and bigger individual crystals were observed already after 30 min. This indicates that Cu-BTC crystallization reactions in methanol proceed at a substantially slower rate compared to that in water.

Due to the use of Na-BTC and BTC in water and methanol, respectively, the main difference next to the solvent is the degree of dissociation of the BTC: in water, the BTC in its salt form is expected to be close to completely dissociated based on its p*K*_a_ values,^41^ while the dissociation of BTC in methanol is expected to be lower, based on the p*K*_a_ difference between water and methanol for benzoic acids.^42^ Analogous to the difference in the dissociation of BTC ions and the zinc ions, it is also possible that not all the copper is fully dissociated in methanol, but to our knowledge, this has not been researched in the literature. Therefore, we hypothesize that the difference in crystal growth for Cu-BTC in water and methanol is mainly governed by the difference in BTC and copper dissociation in these solvents. Furthermore, it has been discussed, for example, by Łuczak^43^ and He et al.,^44^ that different counterions in the same solvent can influence the reactivity of the metal precursor, which can cause a difference in morphology. Therefore, the counterion of the ligand may also play a role in the crystallization process, but a more extensive study is required to confirm the effect of the counterion of the ligand.

The SEM analyses reveal only very limited information about the crystal habit, elemental composition, and other important characteristics of the MOF films. Attempts for more extensive characterization, with other common techniques, also provided only limited additional information. While both EDS in this work (see Figures S2 and S9 in the Supporting Information) and X-ray diffraction of ZIF-8 (see ref (45)) do suggest the formation of the expected MOFs, the results are too substantially influenced by the presence of the underlying membrane to draw sound conclusions. Irrespective of this, our results do clearly demonstrate that our proposed method for the localized formation of a MOF film at the metal-precursor side of an ion exchange membrane is successful and that the localization is due to Donnan exclusion, even in the case when methanol is used as the solvent.

## 4 Conclusions

In this work, we have demonstrated an approach to locally control MOF growth on an AEM via the Donnan exclusion principle for both Cu-BTC and ZIF-8 in water and methanol. However, it is expected that our method can also be applied to any combination of metal and organic linkers that are soluble in water, methanol, or other solvents with relatively high dielectric constants which facilitate ionic dissociation. The benefit of our method compared to the conventional counter-diffusion method for MOF growth is that the synthesis conditions can be tuned for optimal crystallization conditions on the metal reservoir side of the membrane, without the formation of less well-defined crystals. It can also be used to achieve different film thicknesses depending on the nature of MOF, solvent, reaction time, and other experimental parameters.

Next to a promising method to produce MOF films, the method could also be an attractive method to use for composite MOF AEMs. MOF coatings on ion exchange membranes can enhance the selectivity of monovalent versus multivalent ions as well as between different monovalent ions based on their hydration energies during electro-driven separations, such as electrodialysis,^46−48^ offering an alternative to layer-by-layer polyelectrolyte coatings for selectivity^49^ and potentially also leading to enhanced electrodialysis performance if a heterogeneous interface is formed.^50,51^ This method could in principle be used to form MOF films or coatings on ion-exchange membranes within an electrodialysis membrane stack. This would make the process suitable for scale-up to larger membrane areas as well as providing opportunities to repair coating defects by flowing the MOF precursors through a stack.

## Supporting Information Available

Evaluation of the effect of hydroxides on Cu-BTC formation, theoretical dissociation of zinc nitrate in methanol, stability of ZIF-8 precursors in water, and additional SEM results (PDF)
