# Supplementary material for: Controlled Localized Metal–Organic Framework Synthesis on Anion Exchange Membranes
Source: ACS Appl Mater Interfaces. 2024 Jun 10;16(24):31703–8. doi: 10.1021/acsami.4c02882 (PMC11194767; doi:10.1021/acsami.4c02882)
Supplement: Supplementary file 1 — am4c02882_si_001.pdf [file am4c02882_si_001.pdf]

Supporting Information

Controlled Localized Metal Organic Framework Synthesis on Anion Exchange Membranes

Harm T.M. Wiegerinck[1], Özlem H. Demirel[1], Harmen J. Zwijnenberg, Tom van der Meer, Timon Rijnaarts, Jeffery A. Wood,* and Nieck E. Benes*

Membrane Science and Technology Cluster, MESA+ Institute for Nanotechnology, University of Twente, 7500AE Enschede, The Netherlands

E-mail: j.a.wood@utwente.nl; n.e.benes@utwente.nl

[1]Authors contributed equally to this manuscript

S1 Effect of hydroxides on Cu-BTC synthesis

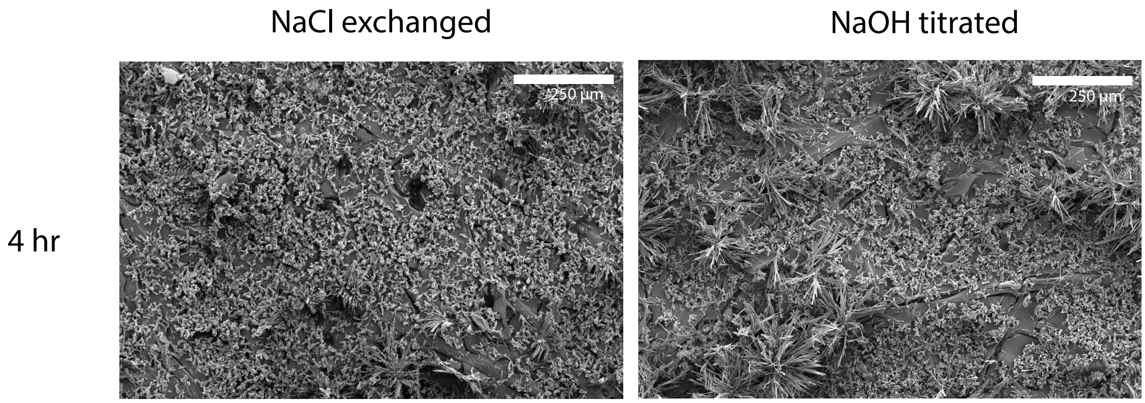

Figure S1: Result of copper nitrate side after 4 hours for a synthesis method where the membrane has been exchanged with sodium chloride and where the Na-BTC has been titrated with NaOH to prevent excess OH^-.

The Cu-BTC synthesis in water presented in the main text used a Na-BTC precursor solution with a pH of 10 due to the addition of a slightly higher than stoichiometric amount of sodium hydroxide based on three acid groups in trimesic acid. Furthermore, the native counterions of the AEM were hydroxide ions. Therefore, it is possible that next to the Cu-BTC copper hydroxide ($Cu(OH)_2$) crystals could have formed on the surface of the AEM, as it is a poorly soluble salt in water.[1] Additionally, the morphology assessed by SEM after 4 hours appeared quite different compared to the morphologies obtained after 24 and 48 hours and this could be related to the formation of copper hydroxide. To try to exclude this effect, we performed additional syntheses of Cu-BTC where we minimized the amount of hydroxide present in both the solution and membrane. This was done using two different synthesis approaches. In the first, only the OH^- counter ions in the membrane were exchanged with chloride ions, and in the second a Na-BTC precursor solution of a pH of 6.8 was prepared by titration in combination with using a membrane in the chloride form.

The results of these two modified synthesis procedures can be seen in Figure S1. In this figure, it can be seen that both the adapted synthesis methods show a similar morphology, while it is clearly different from the original 4-hour synthesis in the main text and shows

similarities to the structures in the main text observed after 24 and 48 hours. This is an indication that hydroxide counter ions in the membrane are most likely the main cause for the morphology observed after 4 hours. This can either be due to the formation of $Cu(OH)_2$ or due to a different morphology of Cu-BTC induced by the local pH as observed in literature.[2]

Due to the similarity between adapted syntheses and the original syntheses after 24 and 48 hours, it seems that the effect of hydroxide counterions only is significant for short synthesis times and is eventually overgrown by a layer of Cu-BTC that is not affected by hydroxide counter ions. Donnan exclusion could also be a reason for the observation that the hydroxide ions from the bulk solution do not affect the Cu-BTC formation substantially: trimesic acid has pKa values of 3.1,3.8 and 4.7.[3] Therefore, at a pH of around 10, a considerable fraction of the trimesic acid is completely dissociated. Due to its higher valency and higher bulk concentration, the trimesic acid will be more enriched in the anion exchange membrane compared to the hydroxide ions, which effectively prevents the hydroxides from transporting through the membrane.

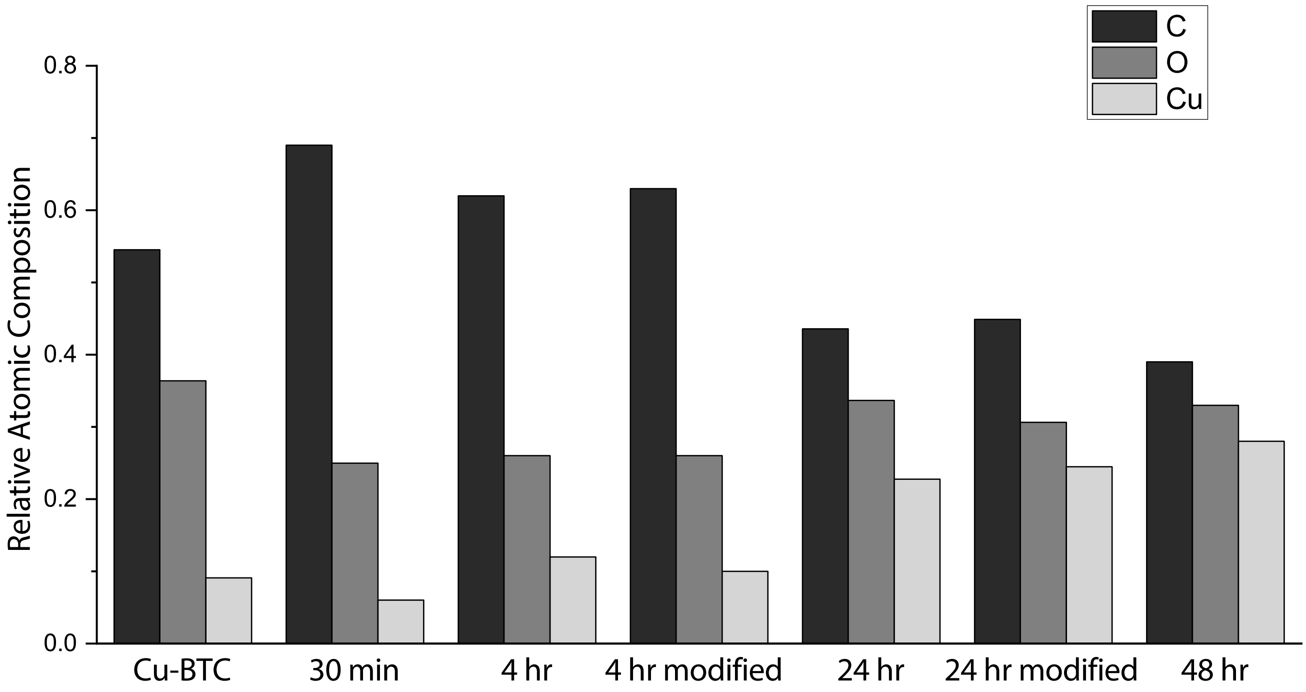

Figure S2: EDS result of the copper side of the AEM for different times and the original synthesis method compared to the second modified synthesis method. In addition, the theoretical elemental composition of the repeating unit of Cu-BTC is shown

To try to elucidate whether the structure observed after 4 hours in the main text is due to $Cu(OH)_2$ formation or due to a morphology change induced by a locally higher pH,[2] the elemental composition of the coating was determined by EDS and represented in Figure S2. The copper content increases with the duration of the synthesis, which is as expected since additional Cu-BTC is formed with longer synthesis times in the SEM images. More interestingly, comparing the original syntheses compared to the titrated syntheses shows almost identical composition results. This also indicates that although the morphology looks quite different after 4 hours, the amount of copper hydroxide that has formed on the surface during the synthesis is most likely minimal and implies that most likely the morphology has been altered locally by local pH changes when comparing a membrane in the hydroxide form or the chloride form.

It should be mentioned that for all the EDS results the theoretical composition is not

retrieved. The primary reason for this is that the membrane underneath the Cu-BTC layer also contains both carbon and oxygen which alters the amount of these elements detected and it is hard to correct for this since it is unknown to what extent the membrane contributes to the signal in all the samples. Based on the ratios of copper and oxygen present, it could be thought that instead of the formation of Cu-BTC, in all cases only copper hydroxide or copper oxide has been formed on the surface. In addition, the morphology of our films is not the typical structure of Cu-BTC particles, which is often shown to have an octahedral structure for example in the work of Najafi Nobar[4] or Nivetha et al.[5] However, typically Cu-BTC is not synthesized using purely water as a solvent, which can have a distinct effect on the crystal structure. Finally, in one of the few cases where only water and precursor is used at room temperature studied by Siddiqui et al.,[6] the crystal structure is more needle-like and seems similar to the Cu-BTC in our SEM results. This, combined with the fact that it is very unlikely that substantial amounts of copper hydroxide or oxide formed during the modified syntheses makes the idea that only copper hydroxides have been formed during our main Cu-BTC syntheses very unlikely.

S2 Dissociation of zinc nitrate in methanol

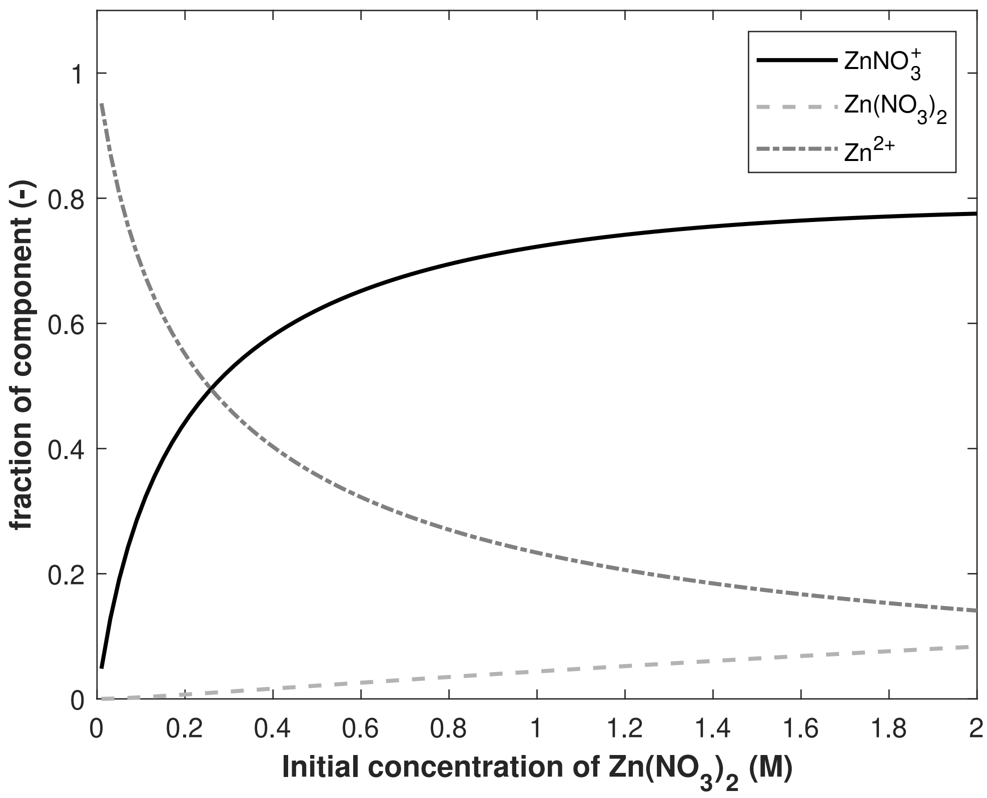

Figure S3: The fractions of different states of zinc nitrate as a function of the $Zn(NO_3)_2$ concentration in methanol. Estimated from the equilibrium constants of Al-Baldawi[7]

In Figure S3 it is shown that theoretically zinc nitrate in methanol primarily dissociates towards $Zn(NO_3)^+$, while there is less than 10 molar percent of the zinc nitrate in the neutral undissociated state up to a concentration of 2 M. This is based experimentally obtained equilibrium constants by Al-Baldawi et al.[7] This also highlights the importance of the charge density of the membrane in methanol, as the membrane could effectively prevent the transport of zinc in methanol, when the charge density and therewith the selectivity is sufficient.

S3 ZIF-8 formation in water

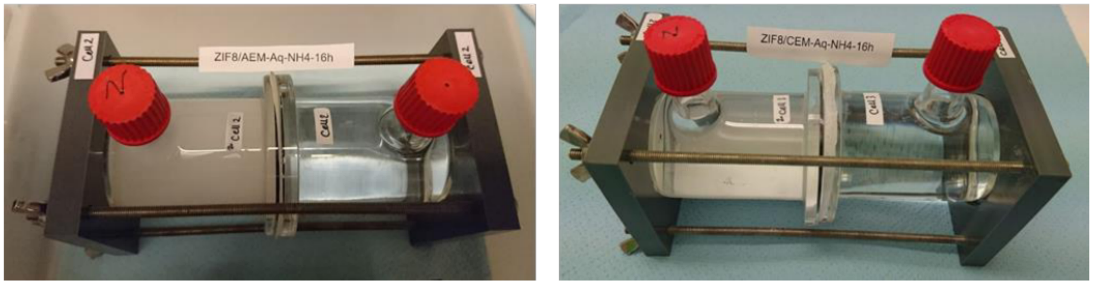

Figure S4: Diffusion cell after 16 hr with zinc nitrate in the left and HMIM with ammonia in the right reservoir for both anion and cation membrane in the left and right figure respectively. Reproduced from PhD thesis of Demirel (2020)[8]

.

Next to the shown experiments in the main text of ZIF-8 in methanol, we also performed the synthesis in water. To make sure that the HMIM was sufficiently dissociated, ammonia was added to increase the pH. During these syntheses, the zinc nitrate reservoir turned opaque, which indicates the formation of insoluble zinc hydroxide. The formation of zinc hydroxide in water during ZIF-8 synthesis was also described by Kida et al.[9] This illustrates that zinc nitrate is an unsuitable precursor in water and that water is not the solvent of choice for localized ZIF-8 synthesis or more generally when the precursors are not stable in water.

S4 Additional SEM results

S4.1 Cu-BTC results

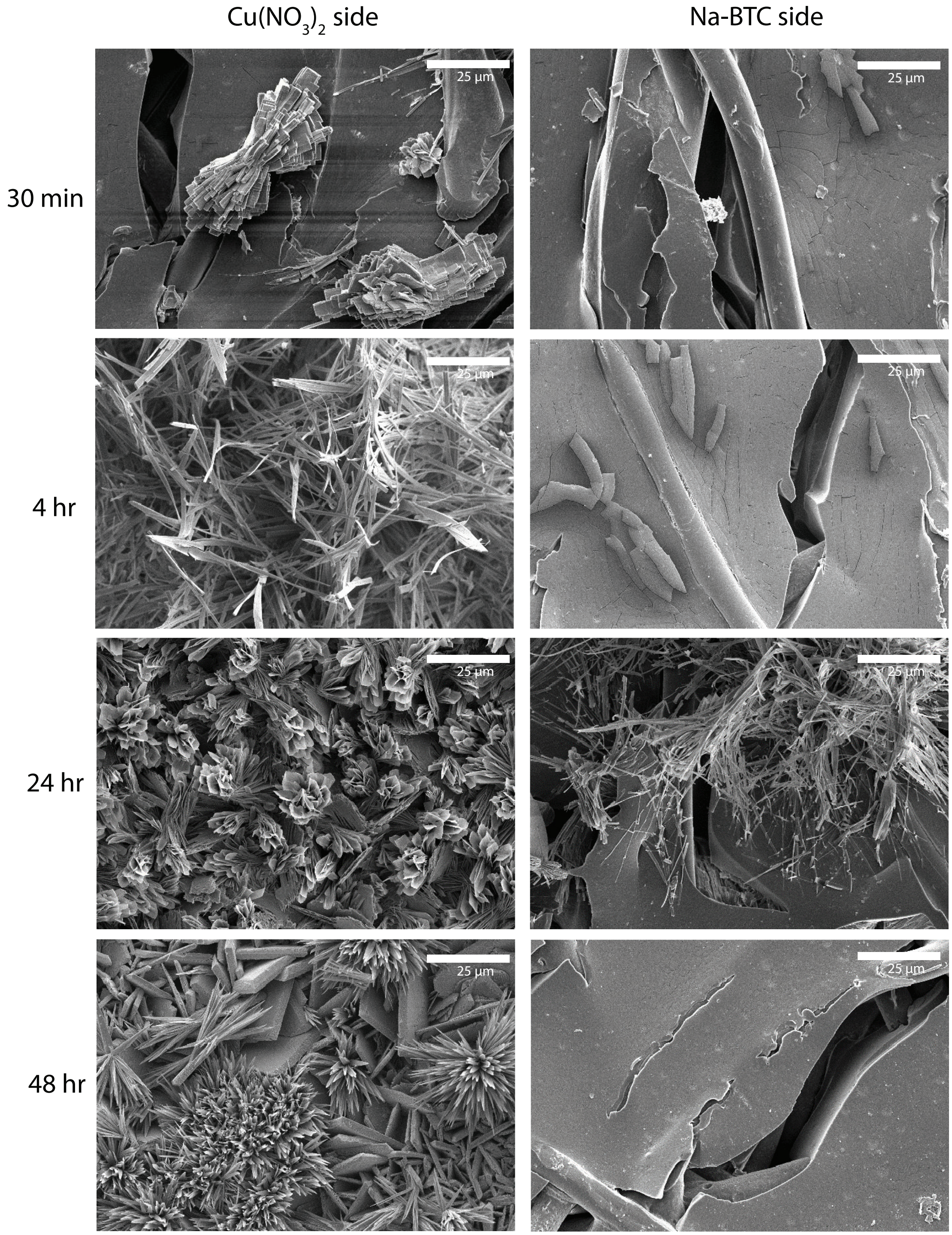

Figure S5: SEM images of the morphology of the surface of anion exchange membranes on the copper nitrate side and the Cu-BTC side, for different times, magnified 1000 times.

Figure S5 shows a close-up of the Cu-BTC coating presented in the main text. In this figure, it can be seen that there is hardly any Cu-BTC present on the Na-BTC side of the membrane, except for a minor spot after 24 hours. However, since there is nothing present after 48 hours, it may be some contamination of the sample. On the copper nitrate side, it can be seen that the morphology goes through various stages, depending on the synthesis time. At 4 hours a needle structure is observed, which is believed to be a change in morphology induced by pH, as described in Section S1. While not very apparent in the main text, the morphology seems to progress to a flower structure after 24 hours and at 48 hours the structure of the surface is a mix of plates and another flower-like morphology. While this could indicate a changing morphology, it could mean that under the current conditions, the morphology is not controlled very well, which could lead to a mix of different structures observed after 24 and 48 hours.

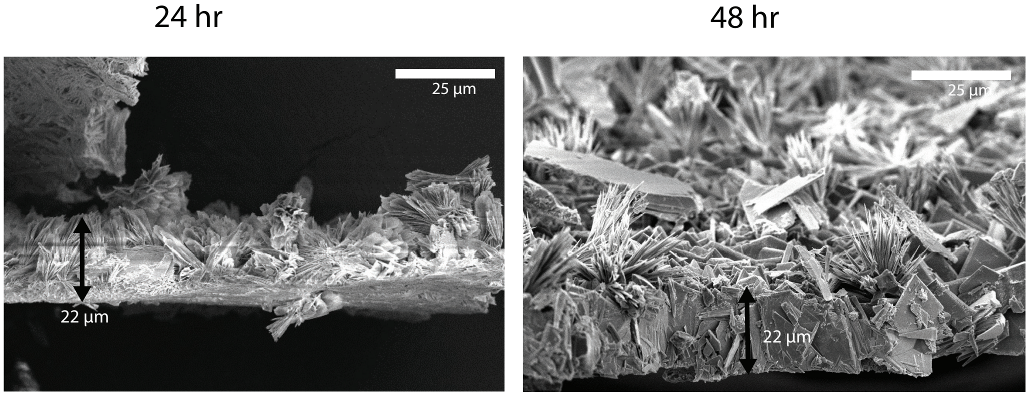

Figure S6: SEM image of a Cu-BTC samples that broke off the anion exchange membrane after 24 and 48 hours.

The thickness was roughly estimated based on small pieces of Cu-BTC coatings that broke off due to the drying stresses of the sample preparation. These SEM images are presented in Figure S6. From Figure S6 it was roughly estimated that the film was about 20 micrometers thick in both cases, indicating that the thickness does not seem to increase beyond 24 hours. A possible explanation for this could be that the Cu-BTC blocks the BTC anions, which prevents further reaction with copper ions to form additional Cu-BTC. In

literature, it is shown that higher valency ions have more difficulty passing the MOF by a combination of size exclusion and the dehydration energy of ions. In that case, the transport of BTC will be increasingly hindered by the growth of the MOF layer over time, resulting in a decreasing growth of the layer thickness.

S4.2 ZIF-8 results

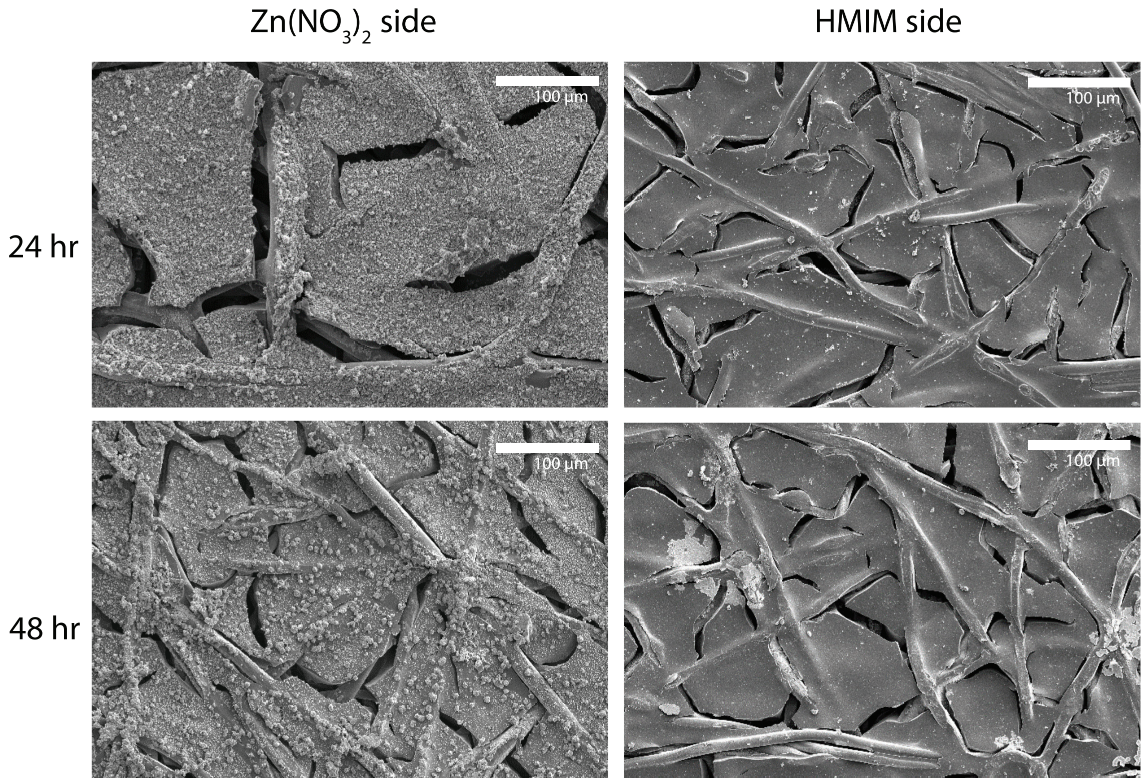

Figure S7: SEM images of the morphology of the surface of anion exchange membranes on the zinc nitrate side and the HMIM side, for different times, magnified 250 times in methanol.

In Figure S7 the ZIF-8 coating discussed in the main text is shown at a lower magnification. This figure shows also that effectively there is only ZIF-8 coating on the zinc nitrate side, with trace amounts of crystals on the HMIM side. Furthermore, this highlights that the coating is quite thin, as the reinforcement fibers are still clearly visible on the coated side.

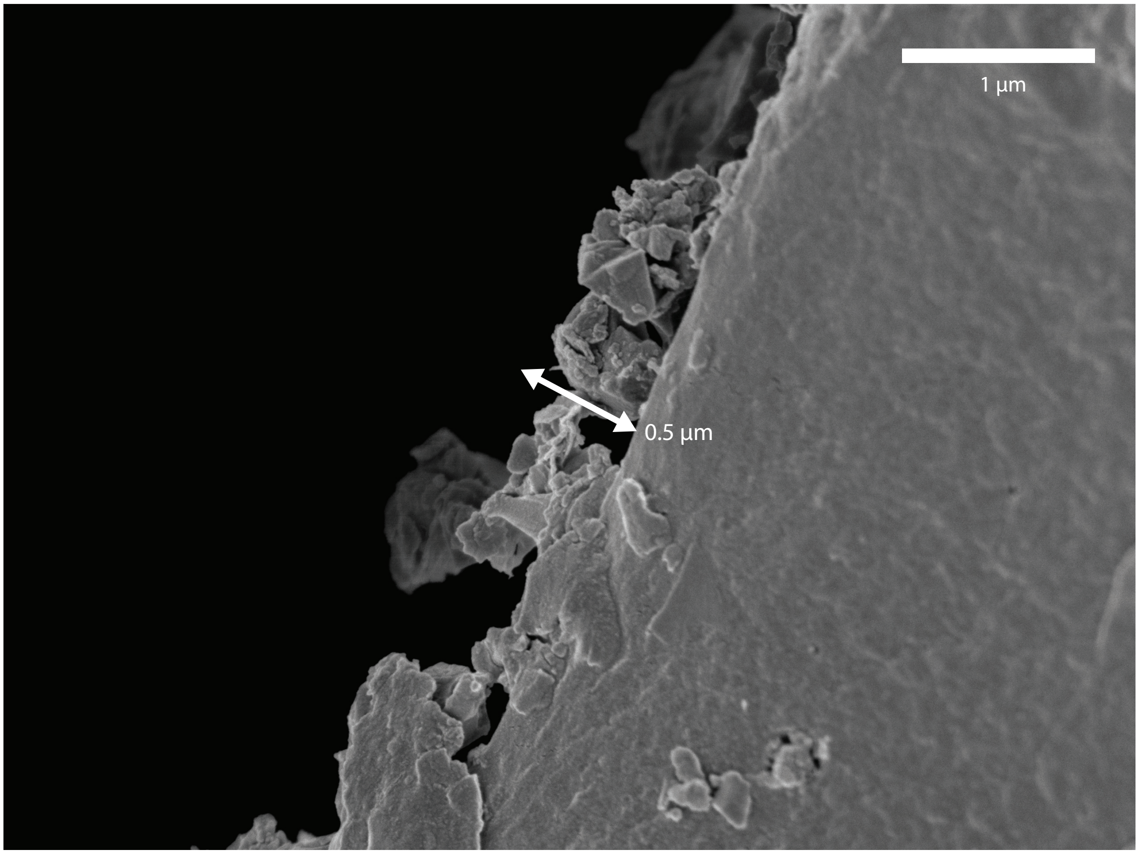

Figure S8: SEM image of a cross-section of an anion exchange membrane, showing the zinc nitrate side of the membrane after a synthesis of 24 hours, magnified 25000 times.

Since the ZIF-8 coating were quite thin, to estimate the thickness of the coating a cross-section by cutting the membrane with a blade, which is shown in Figure S8. While the surface is relatively empty with, due to the cutting as well as the drying of the membrane, it still can be used to estimate the thickness of this coating after a synthesis of 24 hours. From analyzing this image, the coating thickness was estimated to be 0.5 micrometers thick.

S5 EDS of ZIF-8

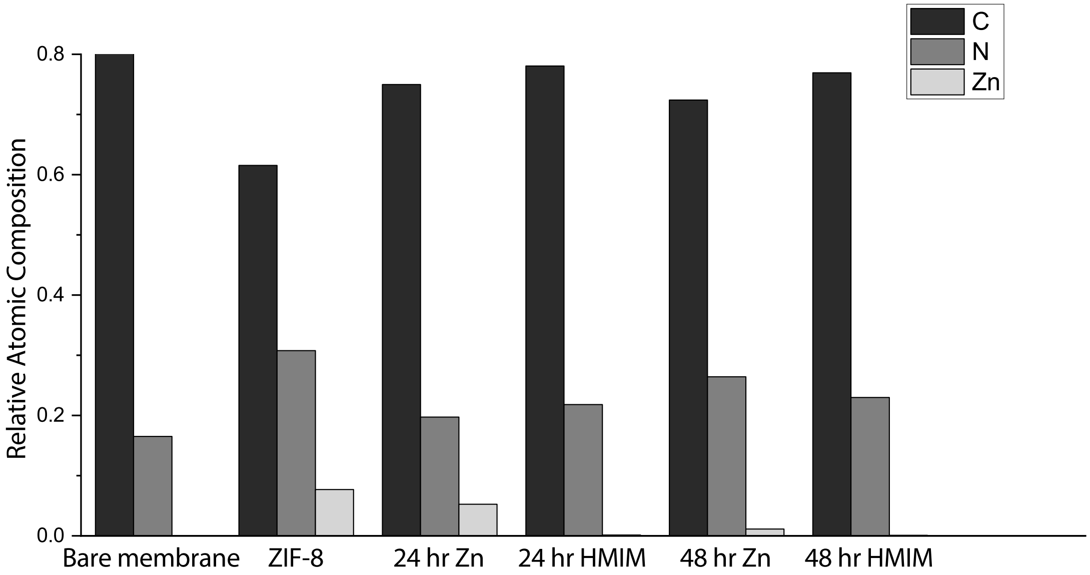

Figure S9: EDS results of the zinc nitrate side and the HMIM side, the bare membrane. In addition, the theoretical elemental composition of the repeating unit of ZIF-8 is shown.

Figure S9, shows that zinc is only found on the zinc nitrate solution side, which is in line with all the SEM images and with the conclusion that the zinc is rejected by the membrane based on Donnan exclusion. Furthermore, the nitrogen content in all the used membranes is consistently higher compared to the bare membrane. This can be understood by noting that HMIM and nitrate, which contain nitrogen atoms, are both able to soak into the membrane since both compounds are either negatively charged or neutral. However, it should be noted that due to the composition of the underlying anion exchange membrane, both the carbon as well as the nitrogen content are influenced considerably.

S6 Additional photos

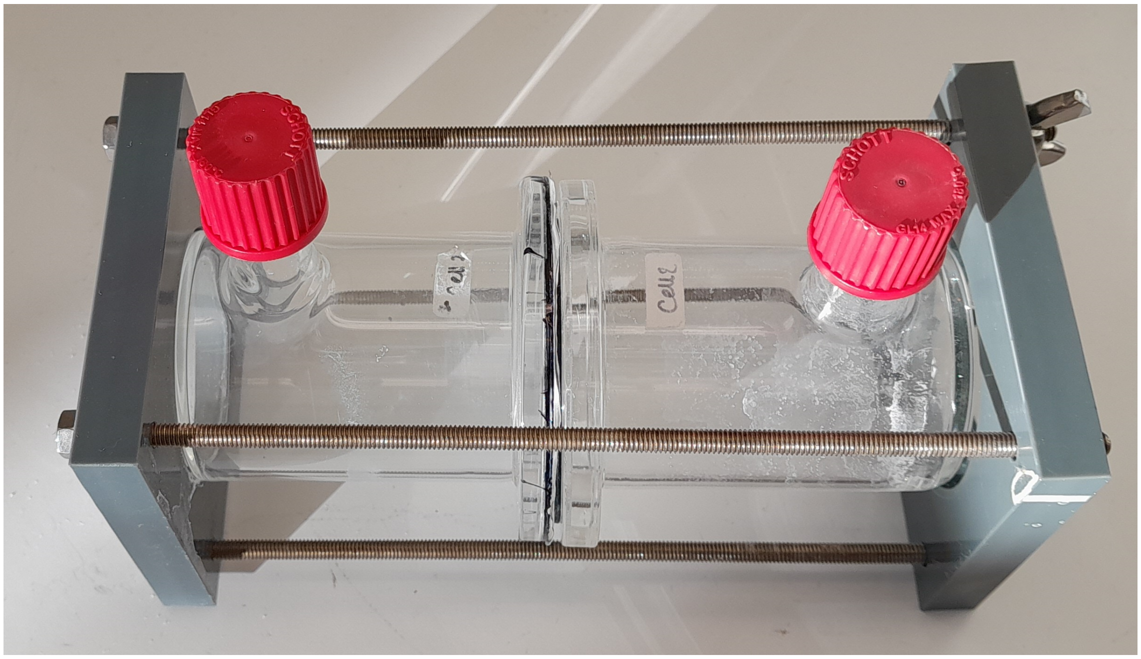

Figure S10: Photo of the used diffusion cell setup.

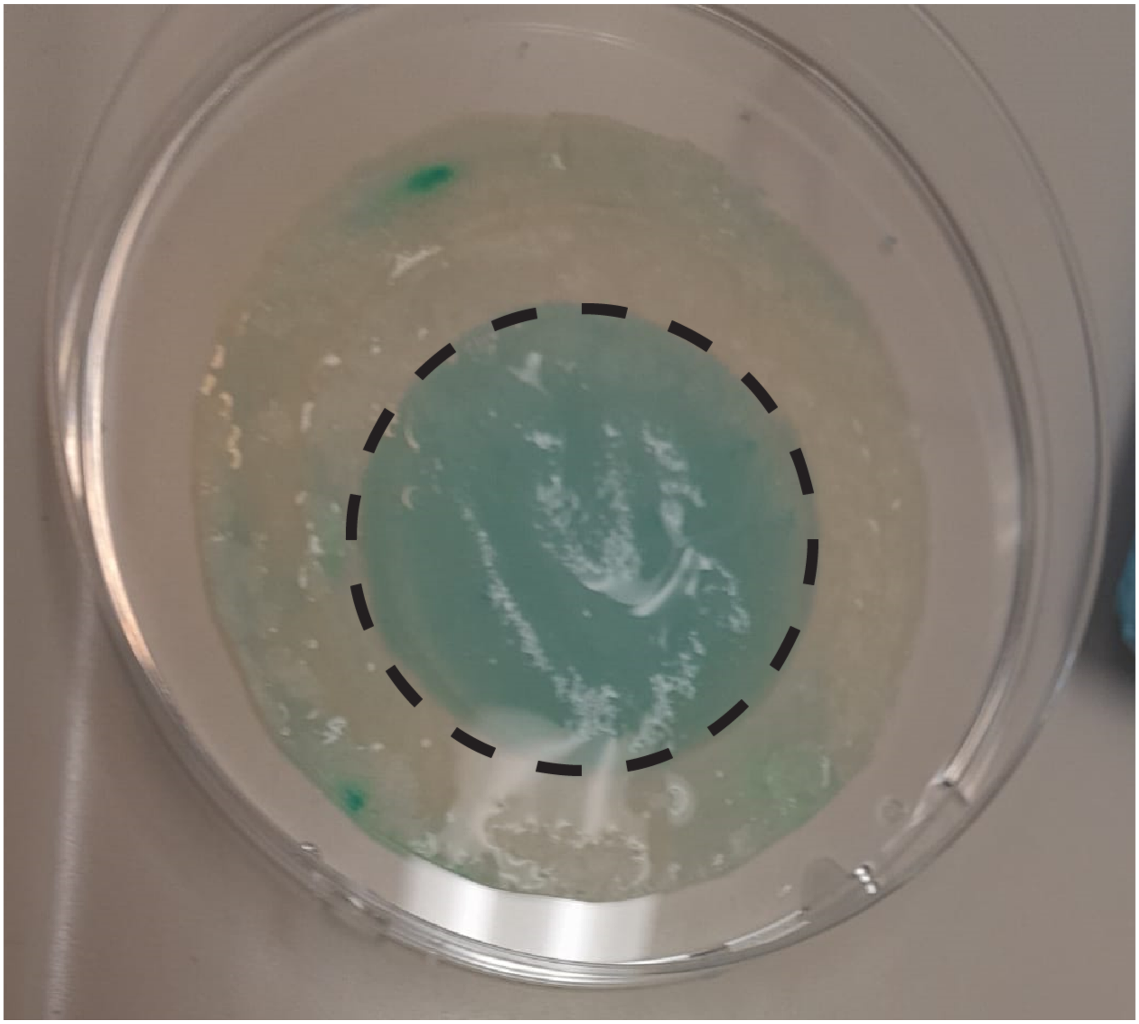

Figure S11: Photo of anion membrane with Cu-BTC on the surface indicated with the marked circle.

References

(1) Dirkse, T. *Copper(II) Oxide and Hydroxide*; 1984; pp 10–26.

(2) Łuczak, J.; Kroczewska, M.; Baluk, M.; Sowik, J.; Mazierski, P.; Zaleska-Medynska, A. Morphology control through the synthesis of metal-organic frameworks. *Advances in Colloid and Interface Science* **2023**, *314*.

(3) Brown, H.; McDaniel, D.; Häfliger, O. *Determination of Organic Structures by Physical Methods*; Elsevier, 1955; pp 567–662.

(4) Najafi Nobar, S. Cu-BTC synthesis, characterization and preparation for adsorption studies. *Materials Chemistry and Physics* **2018**, *213*, 343–351.

(5) Nivetha, R.; Sajeev, A.; Mary Paul, A.; Gothandapani, K.; Gnanasekar, S.; Jacob, G.; Sellappan, R.; Raghavan, V.; Krishna Chandar, N.; Pitchaimuthu, S.; Jeong, S. K.; Nirmala Grace, A. Cu based Metal Organic Framework (Cu-MOF) for electrocatalytic hydrogen evolution reaction. *Materials Research Express* **2020**, *7*.

(6) Siddiqui, S. A.; Prado-Roller, A.; Shiozawa, H. Room temperature synthesis of a luminescent crystalline Cu-BTC coordination polymer and metal-organic framework. *Materials Advances* **2022**, *3*, 224–231.

(7) Al-Baldawi, S. A.; Brooker, M. H.; Gough, T. E.; Irish, D. E. Raman, infrared, and proton magnetic resonance investigation of solutions of zinc nitrate in anhydrous methanol. *Can. J. Chem.* **1970**, *48*, 1202–1208.

(8) Demirel, Ö. H. Metal organic frameworks at interfaces. Ph.D. thesis, University of Twente, Enschede, The Netherlands, 2020.

(9) Kida, K.; Okita, M.; Fujita, K.; Tanaka, S.; Miyake, Y. Formation of high crystalline ZIF-8 in an aqueous solution. *CrystEngComm* **2013**, *15*, 1794.
